# Identification of Enolase as the Target of 2-Aminothiazoles in *Mycobacterium tuberculosis*

**DOI:** 10.3389/fmicb.2018.02542

**Published:** 2018-10-26

**Authors:** Heather H. Wescott, Edison S. Zuniga, Anumita Bajpai, Carolina Trujillo, Sabine Ehrt, Dirk Schnappinger, David M. Roberts, Tanya Parish

**Affiliations:** ^1^TB Discovery Research, Infectious Disease Research Institute, Seattle, WA, United States; ^2^Department of Microbiology and Immunology, Weill Cornell Medical College, New York, NY, United States

**Keywords:** anti-bacterial activity, glycolysis, drug target, tuberculosis, drug target discovery, anti-tubercular

## Abstract

Tuberculosis is a massive global burden and *Mycobacterium tuberculosis* is increasingly resistant to first- and second-line drugs. There is an acute need for new anti-mycobacterial drugs with novel targets. We previously evaluated a series of 2-aminothiazoles with activity against *Mycobacterium tuberculosis*. In this study, we identify the glycolytic enzyme enolase as the target of these molecules using pull down studies. We demonstrate that modulation of the level of enolase expression affects sensitivity to 2-aminothiazoles; increased expression leads to resistance while decreased protein levels increase sensitivity. Exposure to 2-aminothiazoles results in increased levels of metabolites preceding the action of enolase in the glycolytic pathway and decreased ATP levels. We demonstrate that 2-aminothiazoles inhibit the activity of the human α-enolase, which could also account for the cytotoxicity of some of those molecules. If selectivity for the bacterial enzyme over the human enzyme could be achieved, enolase would represent an attractive target for *M. tuberculosis* drug discovery and development efforts.

## Introduction

Tuberculosis (TB) remains a deadly global disease and continues to be one of the leading causes of death worldwide (WHO, 2017). After decades of neglect, there has been a renewed effort in recent years to develop new vaccine and treatment candidates for TB. Drug-sensitive strains can currently be cured with a 6-month combination therapy of isoniazid (INH), rifampicin (RIF), pyrazinamide (PYR), and ethambutol (EMB) for 2 months, followed by INH and RIF for an additional 4 months (Mitchison and Davies, 2012). With the emergence of both multidrug-resistant (MDR) and extensively drug-resistant (XDR) strains, this regimen is becoming increasingly ineffective. Treating MDR and XDR strains of *M. tuberculosis* is very difficult, requiring therapeutic regimens lasting up to 2 years and consisting of more toxic and costly second- and third-line drugs, with often limited success. The discovery and development of new TB drugs with novel mechanisms of action is crucial to our ability to fight this increasing global health threat.

High-throughput screening of diverse chemical libraries has successfully identified a number of compounds with excellent *in vitro* activity against *M. tuberculosis* including the 2-aminothiazoles (2-ATs) (Ananthan et al., 2009). Previous work by our group assessed the structure activity relationships (SARs) of this series to identify compounds with good activity against *M. tuberculosis* and selectivity over mammalian cells (Kesicki et al., 2016). Compounds were rapidly bactericidal and had limited broad-spectrum activity, with some efficacy against *Staphylococcus aureus* and *Mycobacterium smegmatis* (Kesicki et al., 2016). On the basis of these promising findings, we applied a mass-spectrometry (MS)-based proteomics approach to identify the target of 2-ATs in *M. tuberculosis*. The SAR studies completed previously allowed for identification of appropriate residues for coupling the selected small molecule to an affinity matrix with minimal effect on activity of the molecule. We could then utilize this immobilized small molecule to identify the specific interacting protein(s) from a cell lysate. From these studies, we identified enolase as a potential target of 2-ATs in *M. smegmatis*. We used a variety of methods to validate this target in *M. tuberculosis* including both overexpression and underexpression of enolase, metabolomics, measurement of intracellular ATP, and enzymatic activity.

## Materials and Methods

### Microbial Strain Description and Culture Details

*M. tuberculosis* strains used are summarized in Table 1. *M. tuberculosis* was grown in Middlebrook 7H9 medium containing 10% v/v OADC (oleic acid, albumin, dextrose, catalase) supplement (Becton Dickinson) and 0.05% w/v Tween-80. Hygromycin B (Roche) was added to 50 μg/mL, anhydrotetracycline (Alfa Aesar) to 150 ng/mL or 500 ng/mL as indicated, zeocin (Alfa Aesar) to 25 μg/mL, and streptomycin (Sigma) to 20 μg/mL as required. *M. smegmatis* was cultured in Sauton’s medium supplemented with 0.05% Tween-80 pH 7.0.

**Table 1:** Bacterial strains used in this study.

Strain	Relevant characteristics	Reference
*Mycobacterium tuberculosis* H37Rv LP	Wild type	ATCC 25618
Eno-OE	H37Rv LP with vector overexpressing Rv1023; hygR	Melief et al., 2018
*Mycobacterium tuberculosis* Erdman	Wild type	ATCC 35801
Eno-UE	Erdman expressing eno::hyg/pGMCZ-0X-Ptb210eno-DAS/pGMCgS-Teton	This study
*Mycobacterium smegmatis* mc^2^155	Wild type	ATCC 700084

### Generation of a Functional 2-Aminothiazole Affinity Matrix

#### Methyl 4-Hydroxybenzoate


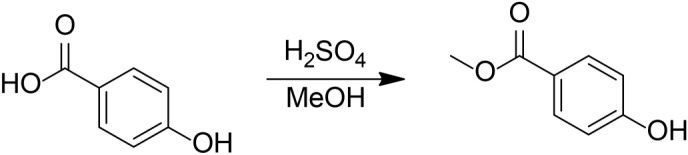


Concentrated sulfuric acid (1 mL) was added to a solution of 4-hydroxybenzoic acid (1.0 g, 7.2 mmol) in methanol (7 mL). The resulting mixture was refluxed for 2 h. The reaction mixture was cooled to room temperature, diluted with 3 M NaOH (aq), let stand for 10 min, then extracted with three portions of ethyl acetate. The combined organic layers were dried over Na_2_SO_4_ and concentrated *in vacuo* to give 150 mg (13%) white solid.

#### Methyl 4-((2-Azidoethoxy)Triethyleneglycol)Benzoate


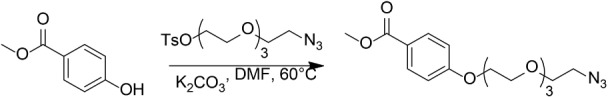


Potassium carbonate (0.3 g, 2.2 mmol) was added to a solution of methyl 4-hydroxybenzoate (0.12 g, 0.79 mmol) and 2-azidoethoxytriethyleneglycol 4-methylbenzene sulfonate (0.45 g, 1.2 mmol) in *N,N*-dimethylformamide (2 mL). The resulting suspension was stirred at 60°C for 24 h. The reaction mixture was cooled to room temperature, diluted with water, and extracted with two portions of ethyl acetate. The combined organic layers were dried over Na_2_SO_4_ and concentrated *in vacuo*. The crude residue was purified by flash column chromatography on silica gel to give 234 mg (84%) colorless oil.

#### 4-((2-Azidoethoxy)Triethyleneglycol)Benzoic Acid


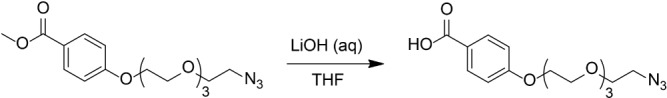


Lithium hydroxide monohydrate (0.14 g, 3.3 mmol) in water (5 mL) was added to a solution of methyl 4-((2-azidoethoxy)triethyleneglycol)benzoate (180 mg, 0.51 mmol) in tetrahydrofuran (5 mL). The resulting mixture was stirred at room temperature for 3 days. The pH of the reaction mixture was adjusted to approximately 2 with dilute HCl (aq), and the mixture was extracted with three portions of ethyl acetate. The combined organic layers were dried over Na_2_SO_4_ and concentrated *in vacuo* to give 161 mg (93%) white solid.

#### 4-((2-Azidoethoxy)Triethyleneglycol)-*N*-(4-Pyridin-2-yl)Thiazol-2-yl)Benzamide


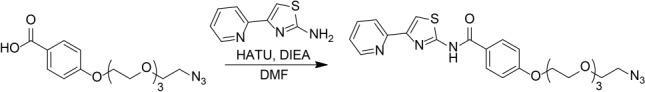


*N,N*-Diisopropylethylamine (80 μL, 0.46 mmol) was added to a solution of 4-((2-azidoethoxy)triethyleneglycol)benzoic acid (100 mg, 0.29 mmol), 2-amino-4-(2-pyridyl)thiazole (52 mg, 0.29 mmol), and HATU (168 mg, 0.44 mmol) in *N,N*-dimethylformamide (2 mL). The resulting solution was stirred at room temperature for 16 h. The reaction mixture was diluted with water and extracted with three portions of ethyl acetate. The combined organic layers were washed with brine, dried over Na_2_SO_4_, and concentrated *in vacuo*. ^1^H NMR showed a mixture of product and starting material. The crude residue was dissolved in ethyl acetate, washed sequentially with 1M HCl (aq), water, 1M NaOH (aq), water, and brine, dried over Na_2_SO_4_, and concentrated *in vacuo* to give 65 mg (45%) yellow oil.

#### 4-((2-Azethoxy)Triethyleneglycol)-*N*-(4-Pyridin-2-yl)Thiazol-2-yl)Benzamide


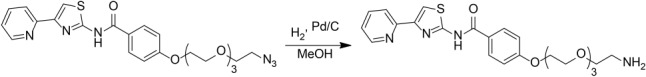


4-((2-Azidoethoxy)triethyleneglycol)-*N*-(4-pyridin-2-yl)thiazol-2-yl)benzamide (48 mg, 96 μmol) was dissolved in methanol in a pressure flask. Palladium on carbon (5 wt%) (4.8 mg, 25 μmol) was added, the flask was evacuated and back-filled to 1 atm with hydrogen three times, and the mixture was shaken on a Parr shaker for 16 h. The reaction flask was evacuated and back-filled with air. The reaction mixture was filtered through a celite plug, washing with methanol. The filtrate was concentrated *in vacuo* to give 42 mg yellow oil. The crude material was used without further purification.

#### 4-((2-Azethoxy)Triethyleneglycol)-*N*-(4-Pyridin-2-yl)Thiazol-2-yl)Benzamide – AffiGel 10 Resin

AffiGel 10 (Bio-Rad, 0.01 mmol/mL loading) (2 mL, 0.02 mmol) was washed with five portions of cold isopropanol then added to a solution of 4-((2-azethoxy)triethyleneglycol)-*N*-(4-pyridin-2-yl)thiazol-2-yl)benzamide (33 mg, 70 μmol) and triethylamine (0.1 mL, 0.73 mmol) in dimethyl sulfoxide (2 mL). The resulting suspension was vortexed for 24 h, at which point LCMS indicated that the free amine had been consumed. Ethanolamine (45 μL, 0.74 mmol) was added, and the resulting suspension was vortexed for 16 h. The solids were collected by vacuum filtration then washed with three portions of 5 mM sodium azide in PBS to give 0.95 g resin. The resin was resuspended in 7.5 mL 5 mM sodium azide in PBS then stored at 4°C prior to use.


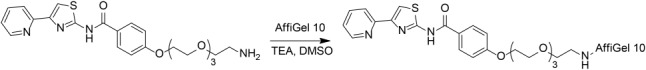


#### Control Resin

To generate a control resin, AffiGel 10 (Bio-Rad, 0.01 mmol/mL loading) (15 mL, 0.15 mmol) was washed with five portions of cold isopropanol then added to a solution of aniline (0.01 mL, 0.11 μmol) and triethylamine (0.2 mL, 1.46 mmol) in dimethyl sulfoxide (6 mL). The resulting suspension was vortexed for 24 h, at which point LCMS indicated that the free amine had been consumed. Ethanolamine (90 μL, 1.48 mmol) was added, and the resulting suspension was vortexed for 24 h. The solids were collected by vacuum filtration then washed with three portions of dimethyl sulfoxide and three portions of 5 mM sodium azide in PBS to give 1.39 g resin. The resin was resuspended in 15 mL 5 mM sodium azide in PBS then stored at 4°C prior to use.


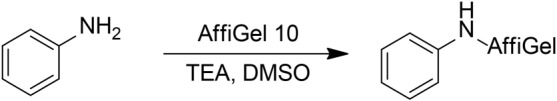


### Identification of 2-Aminothiazole Interacting Proteins by Tandem Mass Spectrometry

The compound and control resins were washed twice with PBS. *M. smegmatis* mc^2^155 was grown in Sauton’s with shaking to an OD_590_ of 0.8. Bacteria were pelleted by centrifugation (4,000 rpm, 10 min), washed 3X with 10 mM Tris pH 8.0, and suspended in 10 mM Tris pH 8.0 + 1X Halt^TM^ Protease Inhibitor Cocktail, EDTA-free (Thermo Scientific). Cells were lysed by bead beating for 60 s. Lysates were cleared by centrifugation at 10,000 rpm for 5 min at 4°C. Protein concentration was determined using BCA Protein Assay (Pierce) and adjusted to 1 mg/mL. 2-AT beads suspension (500 μL) and the same amount of control resin were separately incubated with 20 mg of *M. smegmatis* lysates under continuous rotation (2 h, room temperature). The resins were collected by centrifugation and washed 3X with 10 mM Tris pH 8.0 supplemented with 0.1% Tween-20. Bound proteins were eluted by incubation with 500 μL Tris containing 200 μM unbound IDR-0106878. Elution fractions were concentrated 10-fold using Amicon Ultra filter devices (Millipore) with a 3 kD cutoff. The eluted proteins were separated by SDS-PAGE at 12% acrylamide and stained with Oriole fluorescent gel stain (Bio-Rad). The experiment was repeated twice.

Bands of interest were extracted from both the 2-AT and control lanes of the SDS-PAGE gels and digested using the In-Gel Tryptic Digestion Kit (Thermo Fisher Scientific) according to manufacturer’s instructions. Briefly, gel slices were destained with an ammonium bicarbonate solution. Each slice was reduced with 50 mM TCEP and alkylated with 500 mM iodoacetamide, then washed and rehydrated in trypsin solution at room temperature for 15 min. Protein digestion was allowed to proceed overnight at 30°C with shaking. The peptide samples were desalted using Peptide Desalting Spin Columns (Pierce) and dried.

Desalted peptide samples were resuspended in 10 μL 2% acetonitrile/0.1% formic acid and were analyzed (8 μL) by LC/ESI MS/MS with a Thermo Scientific Easy-nLC II (Thermo Scientific, Waltham, MA, United States) nano HPLC system coupled to a hybrid Orbitrap Elite ETD (Thermo Scientific, Waltham, MA, United States) mass spectrometer. In-line de-salting was accomplished using a reversed-phase trap column (100 μm × 20 mm) packed with Magic C_18_AQ (5-μm 200 Å resin; Michrom Bioresources, Bruker, Billerica, MA) followed by peptide separations on a reversed-phase column (75 μm × 250 mm) packed with Magic C_18_AQ (5-μm 100Å resin; Michrom Bioresources, Bruker, Billerica, MA, United States) directly mounted on the electrospray ion source. A 45-min gradient from 7 to 35% acetonitrile in 0.1% formic acid at a flow rate of 400 nL/min was used for chromatographic separations. The heated capillary temperature was set to 300°C and a spray voltage of 2,250 V was applied to the electrospray tip. The Orbitrap Elite instrument was operated in the data-dependent mode, switching automatically between MS survey scans in the Orbitrap (AGC target value 1,000,000, resolution 240,000, and injection time 250 ms) with MS/MS spectra acquisition in the dual linear ion trap. The 20 most intense ions from the Fourier-transform (FT) full scan were selected for fragmentation in the dual linear ion trap by collisional induced dissociation with a normalized collision energy of 35%. Selected ions were dynamically excluded for 30 s with a list size of 500 and exclusion mass by mass width ±10 ppm.

Data analysis was performed using Proteome Discoverer 1.4 (Thermo Scientific, San Jose, CA, United States). The data were searched against Uniprot Mycobacterium smegmatis with cRAP^1^. Trypsin was set as the enzyme with maximum missed cleavages set to 2. The precursor ion tolerance was set to 10 ppm and the fragment ion tolerance was set to 0.8 Da. Variable modifications included oxidation on methionine (+15.995 Da), and carbamidomethyl on cysteine (+57.021 Da). Data were searched using Sequest HT. All search results were run through Percolator for scoring.

### Construction of *eno* Overexpression Strain

A Gateway compatible entry plasmid bearing the *eno* gene (Rv1023) was obtained from the Pathogen Functional Genomics Resource Center and cloned into the pDTNF expression vector (Galagan et al., 2013) via an LR clonase reaction (Life Technologies). This expression vector contains an anhydrotetracycline (ATc)-inducible promoter and appends an N-terminal FLAG tag on the protein. The plasmid was electroporated into *M. tuberculosis* (Goude et al., 2015) and transformants were selected on plates containing 100 μg/mL hygromycin B (Roche).

### Construction of *eno* Underexpression Strain

Mutants were constructed using methods described previously (Marrero et al., 2010; Schnappinger et al., 2015). Briefly, we first generated a merodiploid strain of *M. tuberculosis* containing a second copy of *eno* (*rv1023*) integrated into the attachment (att) site of the phage L5. We then deleted the native copy of *rv1023* by allelic exchange and replaced the remaining copy of *eno* with an *eno* mutant that contains the DAS+4 degradation tag at the 3′-end and is transcribed by the constitutive promotor P_imyc_ (P_imyc_-eno-DAS) (Kaps et al., 2001; Kim et al., 2011; Schnappinger et al., 2015). The strain containing Pimyc-eno-DAS as the only copy of *eno* was then transformed with plasmids that mediate expression of the DAS+4 recognition protein SspB. Expression of SspB was controlled by a reverse TetR (Klotzsche et al., 2009) so that ATc represses transcription of *sspB*. This resulted in the mutant Eno-TetOn-2 (Eno-UE) in which expression of Eno-DAS is induced by ATc due to the repression of SspB.

### Eno Antiserum Production

Eno rabbit-anti serum was generated by Covance using a full length 6 x His tagged recombinant *M. tuberculosis* protein expressed and purified in *E. coli* using the pET28 expression vector (pET28-eno-his) (Trujillo et al., 2014).

### Determination of Minimum Inhibitory Concentrations

Minimum inhibitory concentrations (MICs) were determined in liquid medium as described previously (Ollinger et al., 2013). Briefly, compounds were solubilized in DMSO and assayed as a 10-point twofold serial dilution series. Bacterial growth was measured by OD_590_ after incubation at 37°C for 5 days. The final DMSO concentration was 2% in all wells. DMSO did not inhibit growth at this concentration. The MIC was defined as the minimum concentration required for complete inhibition of growth. Each experiment had two independent replicates.

### Metabolomics

*M. tuberculosis* was grown in 7H9-OADC-Tw under aerobic conditions in roller bottles at 100 rpm to an OD_590_ of 0.4 prior to addition of aminothiazole or DMSO. Bacteria were harvested and delipidated with chloroform-methanol after 24 h of incubation. Metabolomic and statistical analyses of samples was conducted at Metabolon, Inc. as previously described (Evans et al., 2009; Suhre et al., 2011). Briefly, delipidated *M. tuberculosis* samples (*N* = 6/group) were subjected to methanol extraction. Extracts were split into aliquots and processed for analysis by ultrahigh performance liquid chromatography/mass spectrometry (GC/MS). For LC/MS, the platform was based on a Waters ACQUITY UPLC and a Thermo-Finnigan LTQ mass spectrometer, which consisted of an electrospray ionization (ESI) source and linear ion-trap (LIT) mass analyzer. The sample extract was divided into two aliquots, dried, and then reconstituted in acidic or basic LC-compatible solvents, each of which contained 11 or more injection standards at fixed concentrations. One aliquot was analyzed using acidic positive ion optimized conditions and the other using basic negative ion optimized conditions in two independent injections using separate dedicated columns. Extracts reconstituted in acidic conditions were gradient eluted using water and methanol both containing 0.1% formic acid, while the basic extracts, which also used water/methanol, contained 6.5 mM ammonium bicarbonate. The MS analysis alternated between MS and data-dependent MS2 scans using dynamic exclusion. For GC/MS the samples were re-dried under vacuum desiccation for a minimum of 24 h prior to being derivatized under dried nitrogen using bistrimethyl-silyl-trifluoroacetamide (BSTFA). The GC column was 5% phenyl and the temperature ramp was from 40° to 300°C in a 16-min period. Samples were analyzed on a Thermo-Finnigan Trace DSQ fast-scanning single-quadrupole mass spectrometer using electron impact ionization.

Proprietary software was used to match ions to an in-house library of standards for metabolite identification and metabolite quantification by peak area (Dehaven et al., 2010). For statistical analysis and data visualization, any missing values were assumed to be below the limits of detection. To determine statistical significance, Welch’s Two Sample *t*-tests and two-way ANOVA were used to compare the means of two populations. *P*-values ≤ 0.05 were considered highly significant, *P*-values between 0.05 and 0.1 were considered less significant. An estimate of false discovery rate (FDR) (*Q*-value) was calculated to take into account the multiple comparisons that normally occur in metabolomics-based studies, with *Q* < 0.05 used as an indication of high confidence in a result.

### Measurement of Intrabacterial ATP Levels

*M. tuberculosis* was exposed to compounds for 24 h. ATP levels were measured using the BacTiter-Glo assay kit (Promega). Results are expressed as a percentage of control cells exposed to 2% DMSO. The experiment was repeated twice.

### Measurement of Human α-Enolase Inhibition by 2-Aminothiazole Compounds

Activity of human α-enolase (Sigma) was measured using an indirect coupled pyruvate kinase-luciferase linked assay. Enolase assay buffer comprised of 50 mM HEPES pH 7.0, 7.7 mM KCl, 10 mM MgSO_4_, 0.5 mM D (+)-2-phosphoglyceric sodium salt (2-PG), 1 mM ADP, and 10 units pyruvate kinase (Sigma) was preincubated at 37°C for 3 min. Human α-enolase (Sigma) was added at a concentration of 1 μg per 100 μL of buffer at 37°C. The reactions were incubated for 10 min before measuring ATP production. For measurement of compound inhibition, compounds were added with enolase addition at the indicated concentrations. ATP produced by the reactions was quantified using the ATP Determination Kit (Molecular Probes) according to manufacturer’s instructions. Luminescence was read on a Synergy H1 microplate reader. Experiments were repeated three times in duplicate. For each set of experiments, data was plotted as % inhibition ±standard deviation. ATP produced under the conditions described by α-enolase with 1% DMSO was considered to be 100% active (0% inhibition).

## Results

### Identification of Enolase as Potential 2-AT Cellular Target

**FIGURE 1:**
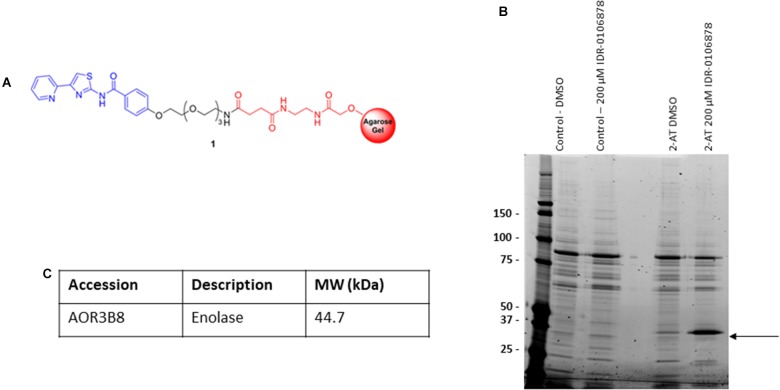
Identification of *M. smegmatis* proteins that bind 2-AT. **(A)** 2-aminothiazole linker resin. **(B)** Pull down assay to identify protein binding. *M. smegmatis* lysates were incubated with the 2-AT and control resins. **(C)** Bound proteins were eluted with excess free compound, or DMSO to identify comparison of non-specific binding. Proteins were analyzed by 1D SDS-PAGE. The indicated band was excised, peptides were extracted and digested with trypsin, followed by mass spectrometry analysis for protein identification.

We were interested in determining the target of a promising series of 2-ATs with potent activity against *M. tuberculosis*. We used a conventional affinity chromatography approach to see if we could identify intracellular targets that bound to the compounds. Our previous work examining structure-activity relationships of these molecules identified an appropriate point for attachment of a resin that would not lead to substantial loss of activity (Kesicki et al., 2016). We crosslinked IDR-0106878 to Affi-Gel 10, an activated matrix that spontaneously couples to amine-containing ligands (Figure 1A). We also synthesized a control resin containing a benzene ring coupled to the Affi-Gel 10 matrix.

We chose to use cell extracts from *M. smegmatis* for our affinity binding assays since 2-AT compounds are active against this organism (Kesicki et al., 2016) and we reasoned that the target in *M. smegmatis* and *M. tuberculosis* was likely to be conserved. We incubated the 2-AT and control resins separately with *M. smegmatis* protein extracts for 2 h at RT to allow interaction between the immobilized compound and its protein binding partner(s). We isolated the matrix beads by centrifugation and washed with buffer to reduce the amount of non-specifically bound proteins associated with the solid matrix. We released the tightly bound proteins by incubation with a high concentration of free compound (IDR-0106878). We resolved the eluted proteins by 1D SDS-PAGE (Figure 1B); the bands enriched in the 2-AT lane relative to the control lanes were excised and subjected to in-gel trypsin digestion. Tryptic peptide mixtures obtained from each gel slice were analyzed by LC-MS/MS, converted into peak lists, and searched using Proteome Discoverer 1.4 against a Uniprot *Mycobacterium smegmatis* database using Sequest HT. The FDR was performed by Percolator. The list of 2-AT potential interactors was refined by superimposition of two independent experiments and removal of the proteins shared with control experiments. From these experiments, we identified the major eluted protein as enolase (Figure 1C) with a high degree of sequence coverage in our samples. The full list of proteins identified as potential 2-AT binding partners can be found in Supplementary Table S1.

### Overexpression of *M. tuberculosis* Enolase Leads to Increased Resistance to 2-AT Compounds

Enolase is predicted to be an essential enzyme, since it plays role in the glycolysis/gluconeogenesis pathway. Therefore it seemed to be a probable target for the series. To validate enolase as a target of 2-AT compounds in *M. tuberculosis*, we generated a strain which overexpressed Eno (Rv1023) (Table 1). We used an ATc-inducible promoter to control *eno* expression (Melief et al., 2018). If Eno is the target of 2-AT compounds, we expect that overexpression of Eno would make the bacteria more resistant to these compounds. We determined MICs for several 2-AT compounds against both the wild-type and Eno-overexpressing (Eno-OE) strain under both inducing and non-inducing conditions (Table 2).

**Table 2:** Enolase overexpression reduces the potency of 2-ATs in *M. tuberculosis.*

Compound^∗^	Wild-type No ATc	Wild-type Plus ATc	*Eno* OE No ATc	*Eno* OE Plus ATc
IDR-0106878	5.1	3.4	40	18
IDR-0106967	3.6	3.6	12	13
IDR-0392806	2.0	1.8	4.5	5.8
IDR-0261770	3.8	3.1	48	17
Rifampicin	0.0053	0.0057	0.0081	0.0081

*^∗^Minimum inhibitory concentrations (μM) were determined against each strain in liquid medium. H37Rv ATCC 25618 was the parental strain. ATc was added at 150 ng/mL to induce expression of *eno* from the plasmid where noted*.

We observed shifts, which varied from 2- to 12-fold for the 2-AT compounds tested. Interestingly we observed a shift under both inducing and non-inducing conditions for the Eno-OE strain. The ATc promoter is known to be leaky, giving rise to low level expression even under non-induced conditions, suggesting that a small amount of additional Eno is sufficient to provide some level of resistance. After induction by ATc, the shifts were generally lower, which might reflect the toxicity of over-expression of a key glycolytic enzyme. This shift in MIC was specific to incubation with 2-AT compounds, as the rifampicin MIC was unchanged between the wild-type H37Rv LP and Eno-OE strains.

### Underexpression of *M. tuberculosis* Enolase Results in Sensitivity to 2-AT Compounds

To further examine the relationship between the level of *eno* expression and effectiveness of 2-AT compounds in inhibiting growth of *M. tuberculosis*, we used a strain engineered to turn off expression of *eno* in the presence of ATc. Just as overexpression of a potential drug target can lead to resistance to that drug, underexpression of the target can lead to greater susceptibility to growth inhibition in the presence of a drug.

In the Eno-UE strain, the native *eno* gene was deleted and replaced with *eno*-DAS under the control of a constitutive promoter expressed from a chromosomally integrated plasmid in the attL5 site. In this strain an adaptation of the *E. coli* SsrA degradation signal is used to control protein stability in mycobacteria (Kim et al., 2011). Expression of the adaptor protein SspB, which recognizes the DAS tag and helps deliver tagged proteins to the protease ClpXP, strongly decreases the activity and protein level of Eno in this strain. SspB is repressed in the presence of ATc, so that removal of ATc leads to degradation of Eno in the underexpressing strain Leaky-eno-tetON. We determined the MIC for several 2-AT compounds for both the wild-type Erdman strain and the under-expressing strain (Eno-UE) in conditions where Eno was expressed at levels close to wild-type with the addition of ATc or where SspB expression was induced so that Eno was degraded in the absence of ATc (Table 3).

**Table 3:** Enolase underexpression results in increased susceptibility to 2-ATs.

Strain^∗^			
**Compound**	**Wild-type No ATc**	**Eno-UE Plus ATc**	**Eno-UE No ATc**
IDR-0106877	9.3	3.8	1.6
IDR-0106883	13	6.6	2.7
IDR-0257155	29	25	10
IDR-0258238	14	13	3.5
IDR-0257156	15	17	2.4
Rifampicin	0.028	0.033	0.022

*^∗^Minimum inhibitory concentrations (μM) were determined against each strain in liquid medium. Erdman ATCC 35801 was the parental strain. ATc was added at 500 ng/mL to repress expression of *sspB* from the tetracycline-inducible promoter where noted, resulting in levels of Eno expression close to wild-type*.

The MICs for each compound were comparable between the wild-type, parental stains (Erdman) and the Eno-UE strain in the presence of ATC, where Eno levels are close to wild-type. However, when Eno levels were reduced (Supplementary Figure S1), we observed increased potency of 2-ATs with a threefold to sixfold shift below wild-type, confirming that lower levels of Eno lead to increased sensitivity. This shift in MIC is specific to 2-AT compounds, as the rifampicin MIC was unchanged between the Erdman wild-type strain and the Eno-UE strain under either inducing or non-inducing conditions.

Taken together, the data demonstrating increased resistance for Eno over-expression and increased sensitivity for Eno under-expression support the identification of enolase as the direct target of 2-AT action.

### Treatment With 2-AT Leads to Increased Levels of Metabolites in Glycolysis Upstream of Enolase

**FIGURE 2:**
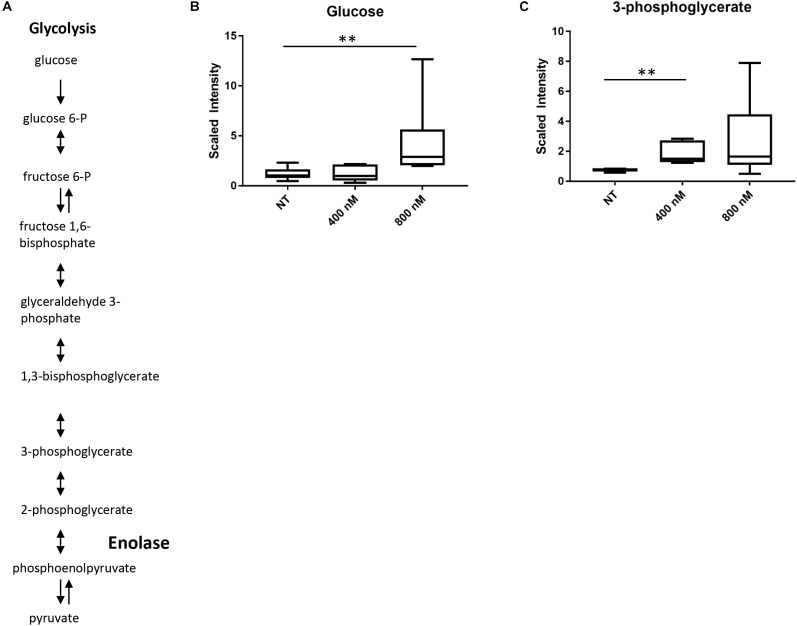
Treatment with 2-AT leads to increased levels of metabolites in glycolysis upstream of enolase. **(A)** Glycolysis pathway. Enolase is responsible for the second to last step of glycolysis, catalyzing the reversible conversion of 2-phosphoglycerate to phosphoenolpyruvate. **(B)** Increased glucose in 2-AT treated cells. Treatment with IDR-0106967 leads to a dose-dependent accumulation of glucose. **(C)** Increased 3-phosphoglycerate in 2-AT treated cells. Treatment with IDR-0106967 leads to a dose-dependent accumulation of 3-phosphoglycerate. *M. tuberculosis* cultures were exposed to 400 nM and 800 nM 2-AT. Data was normalized according to total protein concentration. Box plots show levels of metabolites present in H37Rv plus NT- DMSO; 400 nM – 2-AT; 800 nM – 2-AT. Data are the average ± SD of six replicates. Welch’s Two-Sample *t*-test was used to identify biochemical that differed significantly between groups, ^∗∗^ indicates *p* < 0.01.

To gain a better understanding of the global effects of 2-aminothiazole activity at a biochemical level, we undertook a metabolomic analysis of *M. tuberculosis* in the presence of sub-lethal concentrations of IDR-0106967. Drug treatment was associated with changes in energy metabolism, glucose metabolism, and metabolism of cell wall precursors (Supplementary Table S2). Enolase catalyzes the conversion of 2-phosphoglycerate to phosphoenolpyruvate, the second to last step in the glycolysis pathway (Figure 2A) and several intermediates in glycolysis were significantly altered by drug treatment. Most notably, there was a significant increase in the amount of 3-phosphoglycerate in drug-treated cells compared to untreated control cells (Figure 2C). Alteration of glucose metabolism as a result of 2-AT treatment is further supported by significantly increased glucose levels in *M. tuberculosis* cells treated with 800 nM IDR-0106967 (Figure 2B). These results are consistent with the inhibition of enolase activity by 2-AT compounds resulting in the accumulation of upstream intermediates.

### 2-ATs Deplete Intracellular ATP Levels in *M. tuberculosis*

**FIGURE 3:**
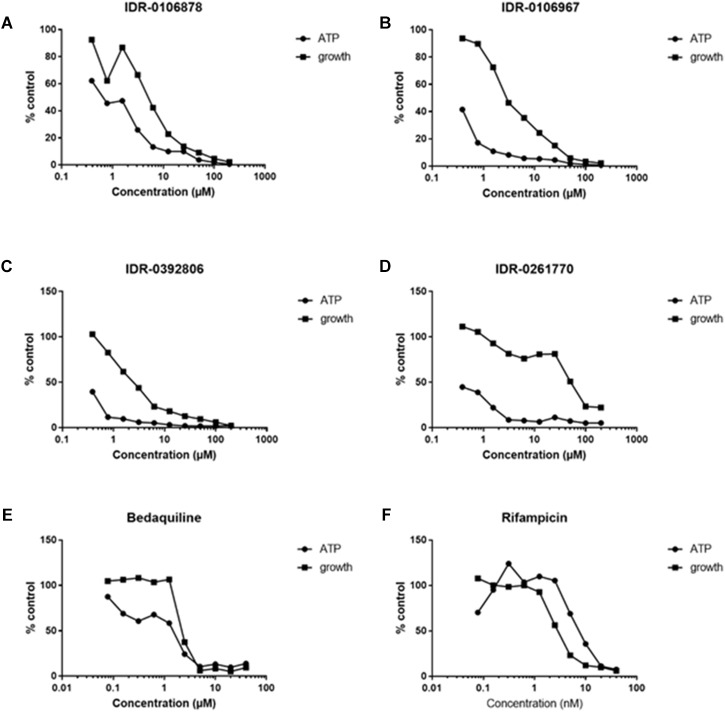
The 2-ATs deplete ATP in *M. tuberculosis*. ATP levels were measured in *M. tuberculosis* H37Rv exposed to compounds for 24 h. Growth after 5 days was measured by OD_590_. **(A–D)** Four 2-AT molecules were tested for their ability to deplete ATP in *M. tuberculosis*. **(E)** Bedaquiline, known to inhibit ATP synthase, serves as a positive control. **(F)** Rifampicin, an inhibitor of RNA polymerase, serves as a negative control for this assay. Data are expressed as a percentage of the DMSO-treated control. Data are representative of two independent experiments.

We hypothesized that if 2-aminothiazole compounds inhibit enolase activity, we should see an effect on ATP levels within bacterial cells. We measured the ATP levels and bacterial growth of *M. tuberculosis* exposed to several 2-ATs. ATP levels within the bacteria were depleted in a dose-dependent manner for all of the compounds tested (Figures 3A–D). The depletion of ATP occurred at concentrations lower than those required to inhibit growth in a similar fashion to that for bedaquiline (Figure 3E), an inhibitor of ATP synthase. This is consistent with ATP depletion being a cause of growth inhibition. In contrast, depletion of ATP by rifampicin, which targets RNA polymerase, is correlated with growth inhibition (Figure 3F).

### 2-ATs Inhibit Human α-Enolase Enzymatic Activity

**FIGURE 4:**
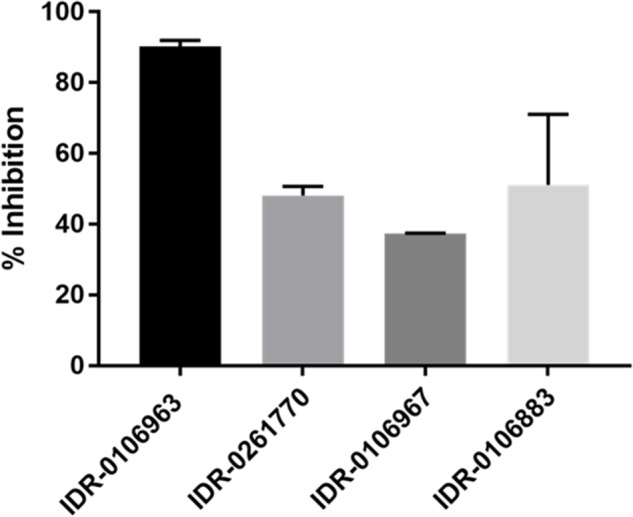
Inhibition of human α-enolase activity by 2-ATs. Human enolase activity was measured using a pyruvate kinase-luciferase linked assay and measured as luminescence. Assays contained 100 μM compound, 0.2 mM 2-phosphoglycerate, and 10 μM enolase. Inhibition was calculated relative to the DMSO-treated control. Data are the mean ± standard deviation for three independent measurements.

We were unsuccessful in our attempts to express and purify active *M. tuberculosis* enolase despite using numerous approaches. Since we were unable to acquire active *M. tuberculosis* enzyme, we decided to evaluate the inhibition of the human α-enolase, which is 49.6% identical at the amino acid level. In addition, since several of the 2-ATs were cytotoxic (Kesicki et al., 2016), we predicted that they would be active against the human enzyme. We tested compounds for their ability to inhibit the human enzyme *in vitro*, and observed enolase inhibition between 35 and 90% with the 2-AT compounds (Figure 4). The most potent compound was IDR-0106963 which inhibited α-enolase activity by greater than 90% under the conditions tested.

## Discussion

Identification of novel drug targets and mechanisms of action are critical for the continued battle against the scourge of TB globally. In this study, we identified enolase as a target for 2-ATs in *M. tuberculosis*. Multiples studies have demonstrated the essentiality of enolase for *in vitro* growth of *M. tuberculosis* using Himar1 transposon mutagenesis (Sassetti et al., 2003; Griffin et al., 2011; Dejesus et al., 2017). Enolase catalyzes the reversible dehydration of 2-phosphoglycerate to phosphoenolpyruvate and participates in both glycolysis and gluconeogenesis. The enzyme is present in a wide variety of organisms and is, like many glycolytic enzymes (Fothergill-Gilmore and Michels, 1993), highly conserved. Recent work suggests that although enolase may be highly conserved across many different organisms, it could be possible to engineer inhibitors with enough specificity to ensure that human cells would not be affected (Navarro et al., 2007).

In this study, we demonstrate that overexpression of enolase in *M. tuberculosis* results in resistance to 2-ATs. This is true even when the plasmid is present but not induced with ATc, so that the amount of additional enolase produced is reduced. This suggests that small changes in the amount of enolase available can have drastic effects on the viability of *M. tuberculosis* in the presence of 2-aminothiazole inhibitors. Conversely, we show that reducing the amount of enolase in the cells by modulating protein stability leads to increased susceptibility of *M. tuberculosis* to 2-ATs. This is likely due to the impact of decreased enolase on glycolysis within the cell leading to more rapid decreases in ATP production, as ATP levels are severely impacted by 2-ATs in wild-type *M. tuberculosis* incubated with 2-ATs.

Here, we demonstrate the inhibition of human α-enolase by 2-ATs. We were interested in determining if there was cross-reactivity between our inhibitors and human α-enolase, since this could be the mechanism of cytotoxicity for these molecules. The lack of a fully active purified *M. tuberculosis* enolase precluded our ability to evaluate their activity against the bacterial enzyme directly. The compounds with inhibitory activity against the human enzyme were also demonstrated to inhibit growth of *M. tuberculosis* and MICs were impacted by the level of enolase expression. This suggests that these molecules are interacting with enolase inside of *M. tuberculosis*. Further work to determine if it is possible to identify 2-aminothiazole analogs that selectively target *M. tuberculosis* enolase over its human counterpart could be beneficial.

Enolase may be most well-known for its role in glycolysis, but much recent work has been focused on its important roles in several other biological and pathophysiological processes. Several glycolytic enzymes are known to be expressed on the surface of some microorganisms (Winram and Lottenberg, 1996; Bergmann et al., 2001; Lahteenmaki et al., 2001; Jones and Holt, 2007; Fulde et al., 2014). These surface localized enzymes are termed moonlighting proteins because of their non-glycolytic functions. Enolase is one of the most widely studied moonlighting proteins in pathogenic microbes, demonstrated to be exported to the surface of *Streptococcus pneumoniae* (Bergmann et al., 2001*), Staphylococcus aureus* (Carneiro et al., 2004), *Bacillus anthracis* (Agarwal et al., 2008), Group A streptococci (Pancholi and Fischetti, 1997), and several others including *M. tuberculosis* (Rahi et al., 2017). For most of these organisms, binding of enolase to host plasminogen has been the primary moonlighting function of enolase that has been characterized. *M. tuberculosis* enolase binds to plasminogen in a lysine-mediated manner (Rahi et al., 2017). Immunization with recombinant enolase was found to provide similar levels of protection in a mouse model of infection to that conferred by immunization with BCG (Horwitz et al., 1995; Brandt et al., 2000; Rahi et al., 2017). Selective mycobacterial enolase inhibitors could be another powerful tool in combating the global health burden of tuberculosis.

## Author Contributions

HW, EZ, DR, and TP: conceptualization. HW, EZ, AB, CT, SE, DS, DR, and TP: methodology and reviewed and edited the manuscript. HW, EZ, and TP: validation. HW and TP: formal analysis and wrote the original draft. HW, EZ, AB, and CT: investigation. TP: supervision. SE, DS, and TP: funding acquisition.

## Conflict of Interest Statement

The authors declare that the research was conducted in the absence of any commercial or financial relationships that could be construed as a potential conflict of interest.

## Footnotes

1 http://www.thegpm.org/crap/
