# Supplementary material for: Identification of Enolase as the Target of 2-Aminothiazoles in Mycobacterium tuberculosis
Source: Front Microbiol. 2018 Oct 26;9:2542. doi: 10.3389/fmicb.2018.02542 (PMC6213970; doi:10.3389/fmicb.2018.02542)
Supplement: Supplementary file 1 [file Data_Sheet_1.PDF]

Supplementary Table S1. Related to Figure 1: Identification of *M. smegmatis* proteins that bind 2-AT

Accession	Description	Score	Coverage	# Sequences	# Unique Sequences
A0R3B8	Enolase_Mycobacterium smegmatis	135	50.8	16	16
A0QS98	Elongation factor Tu_Mycobacterium smegmatis	123	59.9	17	17
A0R203	ATP synthase subunit b-delta_Mycobacerium smegmatis	28.8	24.0	9	9
A0QSD7	30S ribosomal protein S3_Mycobacterium smegmatis	25.4	37.8	8	8
A0R6E3	Uncharacterized protein MSMEG_6518 /MSMEI_6344	25.5	45.7	5	5
A0R7F6	50S ribosomal protein L9_Mycobacterium smegmatis	21.8	53.0	6	6

For each protein, the following parameters are reported: Sequest HT score (Score), percentage of protein sequence covered by matched peptides (Coverage), number of matched peptides (# Sequences) and the number of significant distinct sequence matches in the protein identification process (# Unique Sequences). All proteins with a Sequest HT score below 20 and/or identified with fewer than 5 matched peptides were removed.

Supplementary Table S2. Related to Figure 2: Metabolomics data for *M. tuberculosis* treated with IDR-0106967

			Fold Change		
**Super Pathway**	**Sub Pathway**	**Biochemical Name**	**WT 400nM WT Untr**	**WT 800nM WT Untr**	**WT 800nM WT 400nM**
Amino Acid	Glycine, serine and threonine metabolism	glycine	1.56	0.8	**0.51**
		serine	1.51	0.71	**0.47**
		homoserine	1.55	0.71	**0.46**
		threonine	1.31	0.83	0.63
	Alanine and aspartate metabolism	aspartate	**2.35**	1.51	**0.64**
		beta-alanine	1.25	0.71	**0.57**
		alanine	**1.46**	1.18	0.81
	Glutamate metabolism	glutamate	**1.7**	0.63	0.37
		glutamine	1.33	0.76	0.57
		Gamma-aminobutyrate (GABA)	1.74	0.58	0.34
	Lysine metabolism	diaminopimelate	1.54	0.89	**0.58**
		lysine	1.74	**2.01**	1.16
	Phenylalanine & tyrosine metabolism	phenylalanine	1.31	1.16	0.89
		tyrosine	1.24	0.96	0.78
	Tryptophan metabolism	tryptophan	**1.57**	1.16	0.73
	Valine, leucine, and isoleucine metabolism	levulinate (4-oxovalerate)	**1.57**	1.86	1.19
		beta-hydroxyisovalerate	**1.56**	1.58	1.01
		isoleucine	**1.5**	1.14	0.76
		leucine	**1.51**	1.31	0.87
		valine	**1.61**	1.24	0.77
	Cysteine, methionine, SAM, taurine metabolism	cysteine	1.08	3.26	3.01
		N-formylmethionine	**2.31**	0.66	**0.28**
		S-adenosylhomocysteine (SAH)	**2.29**	0.87	**0.38**
		methionine	**1.96**	**0.59**	**0.3**
	Urea cycle: arginine, proline metabolism	arginine	1.87	0.96	**0.51**
		ornithine	**1.81**	1.51	0.83
		proline	**1.55**	1.07	0.69
		citrulline	**1.72**	0.87	**0.51**
	Polyamine metabolism	5-methylthioadenosine (MTA)	**2.13**	0.65	**0.3**
	Guanidino and acetamido metabolism	4-acetamidobutanoate	**1.59**	**0.62**	**0.39**
	Glutathione metabolism	mycothione (MSSM)	**1.77**	**0.66**	**0.37**
Peptide	Gamma-glutamyl	5-oxoproline	**1.46**	0.83	**0.57**
		gamma-glutamylvaline	**1.89**	1	**0.53**
		gamma-glutamyl-2-aminobutyrate	**1.67**	1.07	0.64
		gamma-glutamylleucine	**1.86**	0.76	**0.41**

		gamma-glutamylisoleucine	**1.76**	0.99	**0.56**
		gamma-glutamylmethionine	**2.72**	**0.26**	**0.1**
		gamma-glutamylglutamine	**1.65**	0.7	**0.43**
		gamma-glutamylphenylalanine	1.48	0.75	**0.51**
		gamma-glutamylthreonine	**1.77**	0.93	**0.52**
		gamma-glutamylalanine	**1.78**	0.69	**0.39**
Carbohydrate	Aminosugars metabolism	erythronate	1.45	1.55	1.07
	Fructose, mannose, galactose, starch and sugar metabolism	fructose	0.68	1.12	1.64
		galactitol (dulcitol)	1.29	**0.56**	**0.43**
		mannose	**1.76**	1.93	1.1
		mannose-1-phosphate	**1.76**	**1.95**	1.11
		mannose-6-phosphate	1.31	0.95	0.72
		sorbitol	0.88	1.18	1.34
		trehalose	1.33	**0.54**	**0.41**
		trehalose-6-phosphate	1.16	1.16	0.99
	Glycolysis, gluconeogenesis, and pyruvate metabolism	glycerate	**1.45**	1.79	1.23
		Glucose-6-phosphate	1.14	1.13	0.99
		Glucose-1-phosphate	1.44	1.43	0.99
		glucose	1	**3.59**	**3.57**
		Frucotose-6-phosphate	1.36	0.89	0.66
		Isobar: fructose 1,6-diphosphate, glucose 1,6-diphosphate, myo-inositol 1,4 or 1,3-diphosphate	1.55	3.38	2.19
		3-phosphoglycerate	**2.41**	**3.54**	1.47
	Nucleotide sugars, pentose metabolism	sedoheptulose-7-phosphate	1.14	0.8	0.7
		gluconate	1.06	0.79	0.75
		Ribose 5-phosphate	1.68	2.13	1.27
		Isobar: ribulose 5-phosphate, xylulose 5-phosphate	0.99	0.77	0.78
		UDP-glucose	1.18	1.45	1.23
		arabinose	0.91	**4.85**	**5.34**
Energy	Krebs cycle	citrate	1.47	1.09	0.74
		2-methylcitrate	1.72	**5.97**	**3.47**
		succinate	1.39	0.89	0.64
		fumarate	**2.16**	2.1	0.97
		malate	**2.26**	1.15	**0.51**
	Oxidative phosphorylation	phosphate	**1.58**	1.46	0.93
		pyrophosphate	2.85	2.81	0.99
Lipid	Medium chain fatty acid	caproate (6:0)	**1.9**	1.44	0.76
		heptanoate (7:0)	**1.68**	**1.64**	0.97
		caprylate (8:0)	**1.6**	1.49	0.93
		pelargonate (9:0)	**1.7**	1.97	1.16
		caprate (10:0)	**1.53**	1.59	1.04
		laurate (12:0)	**1.71**	1.49	0.87

	Fatty acid, monohydroxy	4-hydroxybutarate	**1.74**	1.6	0.92
	Glycerolipid metabolism	ethanolamine	1.76	1.29	0.74
		phosphoethanolamine	2.33	3.13	1.34
		glycerol	**1.7**	1.69	1
		glycerol-3-phosphate	1.41	**0.61**	**0.43**
	Inositol metabolism	myo-inositol	1.37	1.36	1
		inositol-1-phosphate	1.58	1.92	1.22
	Lysolipid	1-palmitoylglycerophosphoethanolamine	1.76	0.91	0.51
		1-palmitoylglycerophosphocholine (16:0)	1.52	1.2	0.79
		1-palmitoylglycerophosphoinositol	**1.75**	1.86	1.06
		1-stearoylglycerophosphoinositol	**1.64**	1.91	1.17
Nucleotide	Purine metabolism, (hypo)xanthine/inosine containing	hypoxanthine	**1.34**	1	0.75
		inosine	1.57	1.87	1.19
	Purine metabolism, adenine containing	adenine	**1.72**	1.29	0.75
		adenosine	1.32	1.46	1.1
		adenosine 5’-monophosphate	1.23	1.22	0.99
		adenosine 5’-diphosphate	**1.73**	2.54	1.47
		adenosine-5’-diphosphoglucose	1.42	3.84	2.7
		adenylosuccinate	0.68	**1.58**	**2.34**
	Purine metabolism, guanine containing	guanine	1.55	**1.78**	1.15
	Pyrimidine metabolism, thymine containing	thymine	1.53	0.91	**0.6**
		thymine 5’-monophosphate	1.56	1.51	0.97
	Pyrimidine metabolism, uracil containing	uridine monophosphate (5’ or 3’)	1.6	1.62	1.01
	Purine and pyrimidine metabolism	methylphosphate	**1.85**	1.89	1.02
Cofactors and vitamins	Ascorbate and aldarate metabolism	Gulono-1,4-lactone	1.3	0.99	0.77
		arabonate	0.88	**3.93**	**4.46**
	Nicotinate and nicotinamide metabolism	nicotinamide	**1.62**	1.58	0.97
		nicotinamide adenine dinucleotide (NAD+)	**2.04**	1.61	0.79
		nicotinamide adenine dinucleotide reduced (NADH)	**1.71**	0.59	**0.35**
		nicotinate	**1.6**	0.94	**0.59**
		nicotinic acid mononucleotide (NaMN)	**1.96**	1.22	0.63
	Pantothenate and CoA metabolism	pantothenate	**1.55**	1.21	0.78
		acetyl CoA	1.48	2.65	1.79
		2,3-dihydroxyisovalerate	1.97	2.04	1.04

	Riboflavin metabolism	flavin adenine dinucleotide (FAD)	**1.71**	1.11	0.65
		flavin mononucleotide (FMN)	**1.81**	0.68	**0.38**
	Vitamin B6 metabolism	pyridoxine (Vitamin B6)	**1.43**	1.75	1.22
Xenobiotics	Benzoate metabolism	benzoate	1.82	1.77	0.97
		4-hydroxybenzoate	**1.63**	1.23	0.75
		p-hydroxybenzaldehyde	1.12	1.66	1.47
	Chemical	2-hydroxyisobutyrate	1.59	1.36	0.85
		triethyleneglycol	**1.74**	**2.25**	1.29
		heptaethylene glycol	**2.37**	**3.58**	1.51
		hexaethylene glycol	**2.43**	**4.08**	1.68
		octaethylene glycol	**2.1**	**3.3**	1.57
		pentaethylene glycol	**2.75**	**4.43**	1.61
		tetraethylene glycol	**2.81**	**4.4**	1.56
		2-ethylhexanoate	**1.58**	0.9	**0.57**
		beta-methyllevulinate	**1.62**	1.61	0.99
	Food component/Plant	4-hydroxybenzyl alcohol	**1.56**	1.02	**0.66**
		ergothioneine	**2.22**	**0.37**	**0.17**

Blue and green shaded cells indicate that p≤0.05 (blue indicates that the mean values are significantly higher for that comparison; green values significantly lower). Light blue and light green shaded cells indicate 0.05<p<0.10 (light blue indicates the mean values trend higher for that comparsion; light green values trend lower).

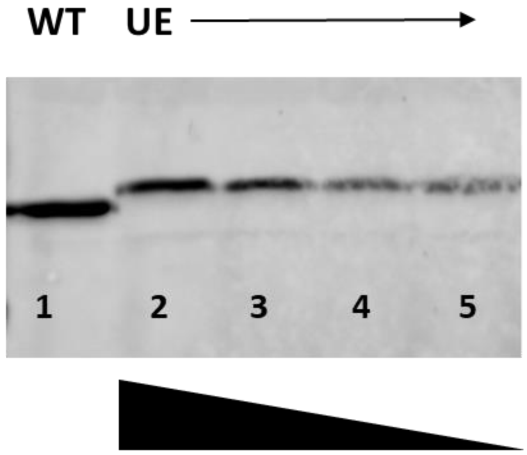

Figure S1. Response of Eno-UE strain to decreasing concentrations of ATC. *M. tuberculosis* Erdman (WT) and Eno-UE (UE) were grown in 7H9-OADC-Tw +/- ATC to exponential phase. The level of Eno produced in the presence of 500 ng/mL (lane 2), 250 ng/mL (lane 3), 125 ng/mL (lane 4) and 0 ng/mL (lane 5) was determined by immunoblotting with α-Eno antibody.
